# Measuring the ratio of femoral vein diameter to femoral artery diameter by ultrasound to estimate volume status

**DOI:** 10.1186/s12872-021-02309-7

**Published:** 2021-10-20

**Authors:** Zhihang Ma, Jiaxin Gai, Yinghan Sun, Yunpeng Bai, Hongyi Cai, Lei Wu, Lixiu Sun, Junyan Liu, Li Xue, Bingchen Liu

**Affiliations:** Department of Cardiology, The Fourth Affiliated Hospital of Harbin Medical University, Harbin, Heilongjiang China

**Keywords:** Femoral vein diameter, Femoral artery diameter, CVP, mPAP, Volume status, Pulmonary hypertension

## Abstract

**Background:**

Currently, the accepted effective method for assessing blood volume status, such as measuring central venous pressure (CVP) and mean pulmonary artery pressure (mPAP), is invasive. The purpose of this study was to explore the feasibility and validity of the ratio of the femoral vein diameter (FVD) to the femoral artery diameter (FAD) for predicting CVP and mPAP and to calculate the cut-off value for the FVD/FAD ratio to help judge a patient’s fluid volume status.

**Methods:**

In this study, 130 patients were divided into two groups: in group A, the FVD, FAD, and CVP were measured, and in group B, the FVD, FAD, and mPAP were measured. We measured the FVD and FAD by ultrasound. We monitored CVP by a central venous catheter and mPAP by a Swan-Ganz floating catheter. Pearson correlation coefficients were calculated. The best cut-off value for the FVD/FAD ratio for predicting CVP and mPAP was obtained according to the receiver operating characteristic (ROC) curve.

**Results:**

The FVD/FAD ratio was strongly correlated with CVP (R = 0.87, *P* < 0.0000) and mPAP (R = 0.73, *P* < 0.0000). According to the ROC curve, an FVD/FAD ratio ≥ 1.495 had the best test characteristics to predict a CVP ≥ 12 cmH_2_O, and an FVD/FAD ratio ≤ 1.467 had the best test characteristics to predict a CVP ≤ 10 cmH_2_O. An FVD/FAD ratio ≥ 2.03 had the best test characteristics to predict an mPAP ≥ 25 mmHg. According to the simple linear regression curve of the FVD/FAD ratio and CVP, when the predicted CVP ≤ 5 cmH_2_O, the FVD/FAD ratio was ≤ 0.854.

**Conclusion:**

In this study, the measurement of the FVD/FAD ratio obtained via ultrasound was strongly correlated with CVP and mPAP, providing a non-invasive method for quickly and reliably assessing blood volume status and providing good clinical support.

## Background

In humans, blood volume is an important factor affecting the stability of haemodynamics, and an imbalance in blood volume can lead to a variety of critical clinical conditions. On the one hand, excess volume can lead to oedema, ascites, and an increase in extracellular volume. On the other hand, insufficient volume can cause multiple organ dysfunction syndrome (MODS). Therefore, assessing volume status is critical for treating disease. Blood volume status can be assessed invasively or non-invasively. There are many invasive assessment methods, such as the measurement of central venous pressure (CVP) and mean pulmonary artery pressure (mPAP). CVP is influenced by a number of factors (including thoracic, pericardial, and abdominal pressures and the specification of operational measurements) [1]. As an indicator of fluid management, CVP sometimes cannot directly reflect blood volume and sometimes may mislead treatment decisions [1, 2]. mPAP is an important index for evaluating pulmonary circulation volume status [3]. It is invasively measured with a pulmonary artery catheter (PAC), which needs to be monitored in an intensive care unit (ICU) and cannot provide fast and effective support for clinical work [4]. Moreover, there are certain risks associated with these invasive methods. Complications related to the use of a PAC include those associated with venipuncture, such as arteriovenous fistula, pneumothorax, and thrombosis; those associated with catheterization, such as arrhythmia; time-related complications of the PAC in the cardiovascular system, such as infection; and incorrect interpretation or use of exported data [4]. Given the above shortcomings of invasive assessment methods, a non-invasive, rapid, and effective method for assessing blood volume status is urgently needed.

Among the non-invasive evaluation methods that have been explored, measuring the inferior vena cava (IVC) diameter with ultrasound is considered a reliable evaluation method with a good correlation with volume status [5–8]. The IVC diameter and its collapsibility index (IVC-CI) can be measured by ultrasound to evaluate CVP [9, 10]. However, the accuracy of measuring the IVC diameter via ultrasound is sometimes affected by certain factors, such as abdominal trauma, increased intra-abdominal pressure, ventricular contraction, obesity, and the patient's body position during measurement [5, 11]. Furthermore, measuring the IVC diameter by ultrasound is not as easy to obtain as that for the superficial vein, and the requirements for surveyors and ultrasonic instruments are higher [6]. mPAP is an important indicator of haemodynamic monitoring and is of great significance for the diagnosis and treatment of some pulmonary diseases. Currently, most non-invasive assessments are performed by echocardiography, but most of them are based on the peak velocity of tricuspid regurgitation; for patients without tricuspid regurgitation, mPAP cannot be measured by this method [12, 13].

In recent years, scholars have explored another non-invasive method for evaluating CVP: measurement of the femoral vein diameter (FVD) by ultrasound [14, 15]. Although experiments have suggested that the FVD has a good correlation with CVP, individual FVDs vary greatly and are affected by age, sex, height, body mass index, and other factors [16]. Therefore, to avoid the influence of these factors, we adopted the FVD/femoral artery diameter (FAD) ratio in this study and explored its correlation with CVP and mPAP.

## Materials and methods

### Study design

This prospective study was conducted in the intensive care unit of the Fourth Affiliated Hospital of Harbin Medical University in China. The study was approved by a hospital committee (ethical approval number: 2021-SCILLSC-10), and informed consent was obtained from the patient or authorized person. The inclusion criteria were as follows: patients over 18 years of age who required haemodynamic monitoring, for example, multiple organ failure, shock, heart failure, myocardial infarction, acute pulmonary oedema, acute pulmonary embolism, etc. The exclusion criteria were as follows: (a) patients with a right atrium or right ventricle tumour; (b) patients with severe stenosis of the pulmonary valve or tricuspid valve; (c) patients with serious malformation of the pulmonary artery; (d) patients with thrombocytopenia or other serious clotting disorders; (e) patients with a skin infection at the puncture site; (f) patients receiving mechanical ventilation; and (g) patients with lower extremity artery/vein thrombosis, significant lower extremity artery plaque, lower extremity artery occlusion, inferior vena cava filter implantation, lower extremity varicose veins, or aortic stenosis.

Two highly trained doctors separately performed puncture and ultrasound examinations to minimize operational errors. The doctors performing the ultrasound examination were unaware of the values of CVP and mPAP. The patient was in the supine position throughout the ultrasound examination and haemodynamic monitoring. We used an EPIQ7 ultrasound machine (Phillips, USA) to measure the FVD and FAD.

First of all, twenty normal subjects were randomly selected, and their FVD and FAD were measured to determine the baseline FVD/FAD ratio. Then, the 130 patients were divided into two groups: Group A included patients requiring central venous catheter placement in ICU, such as shock, right heart failure, long-term infusion or intravenous hypertrophic therapy, etc. Group B included patients requiring Swan-Ganz floating catheter implantation in ICU, such as pulmonary oedema, pulmonary hypertension, and left heart failure, etc. In group A, the FVD, FAD, and CVP were measured. In group B, the FVD, FAD, and mPAP were measured.

### Ultrasound measurement method

**Fig. 1:**
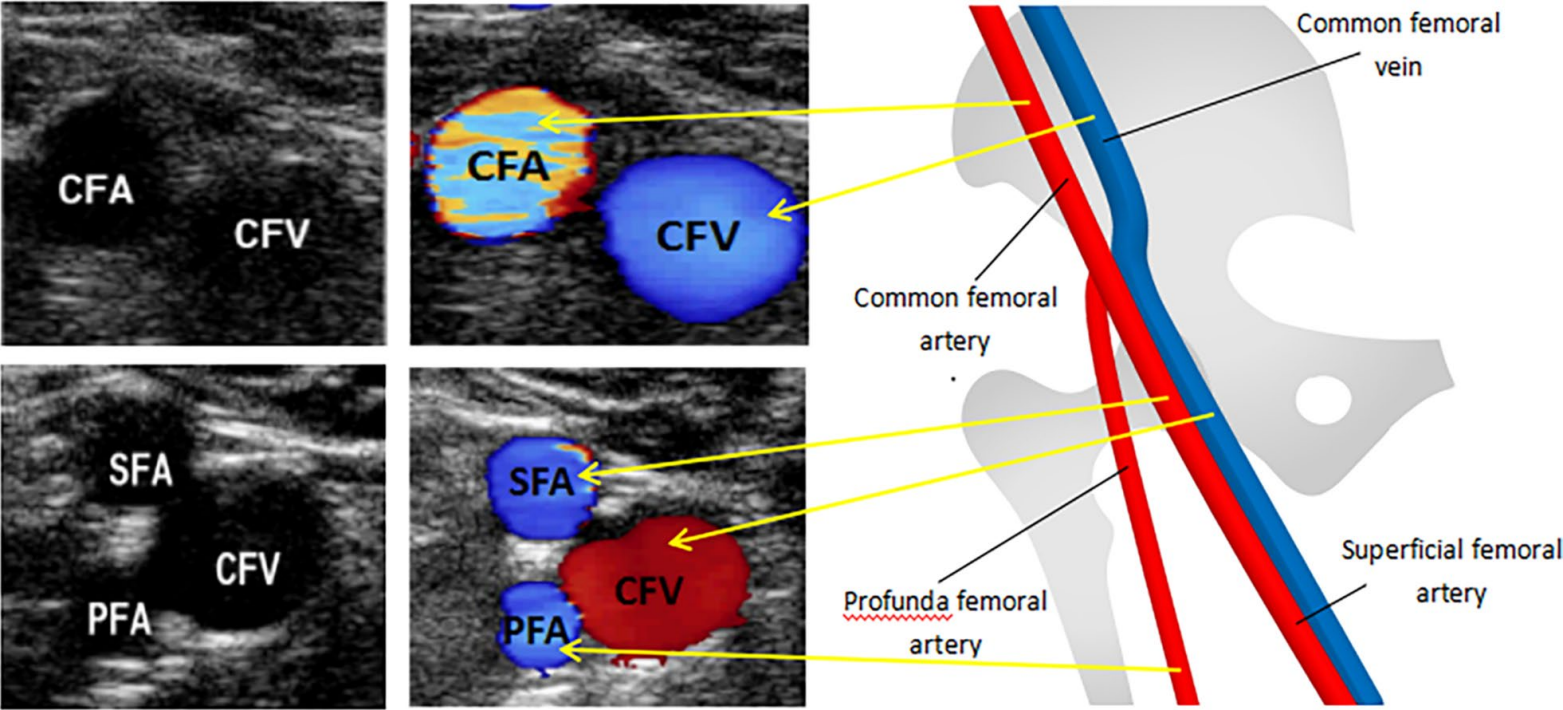
The images of femoral artery and vein were measured by ultrasound

An ultrasound probe was used to first find the bifurcation position of the femoral artery, and then the probe was retracted proximally. The visual field of the bifurcation disappeared until the probe entered the main branch of the femoral artery, and the femoral artery and vein could be observed simultaneously. Under normal conditions, pulsation is an indication of the femoral artery, and its companion is the femoral vein (Fig. 1). The mean FVD and FAD (3–5 diametral lines in different directions) were measured, and then the patient was asked to cough or perform Valsalva manoeuvres. Changes in the femoral vein were observed within half a minute, and the FVD was measured again (mainly used for FVD less than FAD). The FVD/FAD ratio and the FVD/FAD ratio after cough (exFVD/FAD) were obtained.

### Haemodynamic monitoring

After placing the central venous catheter/Swan-Ganz floating catheter through the right internal jugular vein, the pressure sensor was connected at the opening of the central venous catheter/Swan-Ganz floating catheter and was linked to the monitor. The pressure sensor was placed on the level of the patient's axillary midline. After the sensor was zeroed successfully, the values of CVP and mPAP were recorded.

### Statistical analysis

All the data were input and analysed with R 4.0.2 software. The results are expressed as the mean ± standard deviation (SD). *P* < 0.05 was considered statistically significant. Multiple linear regression was used to analyse the relationship between multiple independent variables and dependent variables. The Pearson correlation coefficient was used to analyse the relationship between the FVD/FAD ratio and CVP/mPAP. Simple linear regression was used to analyse the variable dependence. The receiver operating characteristic (ROC) curve was used to find the cut-off value.

## Results

The study included 130 patients. There were 32 males and 33 females in group A and 28 males and 37 females in group B. In group A, the mean age was 65.5 ± 10.2 years, the mean FVD/FAD ratio was 1.54 ± 0.30, and the mean CVP was 13.22 ± 4.11 cmH_2_O, the mean exFVD/FAD ratio for patients with the FVD/FAD ratio ≤ 1 is 1.23 ± 0.10. In group B, the mean age was 67.3 ± 10.3 years, the mean FVD/FAD ratio was 2.00 ± 0.32, and the mean mPAP was 25.63 ± 4.96 mmHg. The mean FVD/FAD ratio of twenty normal subjects was 1.18 ± 0.04. The mean exFVD/FAD ratio of twenty normal subjects was 1.52 ± 0.09.

The FVD/FAD ratio, age and sex were independent variables, CVP and mPAP were taken as dependent variables, and multiple linear regression analysis was performed (Table 1). Analysis of variance (ANOVA) was conducted, and the results are shown in Table 2. Age and sex had no significant effect on CVP and mPAP (*P* > 0.05). The FVD/FAD ratio was an influential factor for CVP and mPAP (*P* < 0.05). Therefore, simple linear regression was carried out with the FVD/FAD ratio as the only independent variable and CVP and mPAP as the dependent variables.

**Table 1:** Multiple regression analysis

	CVP				mPAP			
	Unstandardized Coefficient B	t	*P*	95%CI	Unstandardized Coefficient B	t	*P*	95%CI
Constant	− 3.926	− 1.776	0.081	− 8.346–0.494	4.174	1.203	0.234	− 2.766–11.115
FVD/FAD	11.997	14.184	< 0.00	10.306–13.689	11.647	8.734	< 0.00	8.980–14.313
Age	− 0.027	− 1.081	0.284	0.076–0.023	− 0.039	− 0.943	0.350	− 0.121–0.043
Gender	0.816	1.631	0.108	− 0.184–1.817	1.304	1.563	0.123	− 0.365–2.973

**Table 2:** ANOVA test

	CVP				mPAP			
	Df	Mean square	F	*P*	Df	Mean Square	F	*P*
FVD/FAD	1	820.84	203.1953	< 0.00	1	859.18	77.6362	< 0.00
Age	1	4.80	1.1878	0.2801	1	10.76	0.9727	0.3279
Gender	1	10.75	2.6610	0.1080	1	27.03	2.4421	0.1233
Residuals	61	4.04			61	11.07		
Total	64				64			

**Fig. 2:**
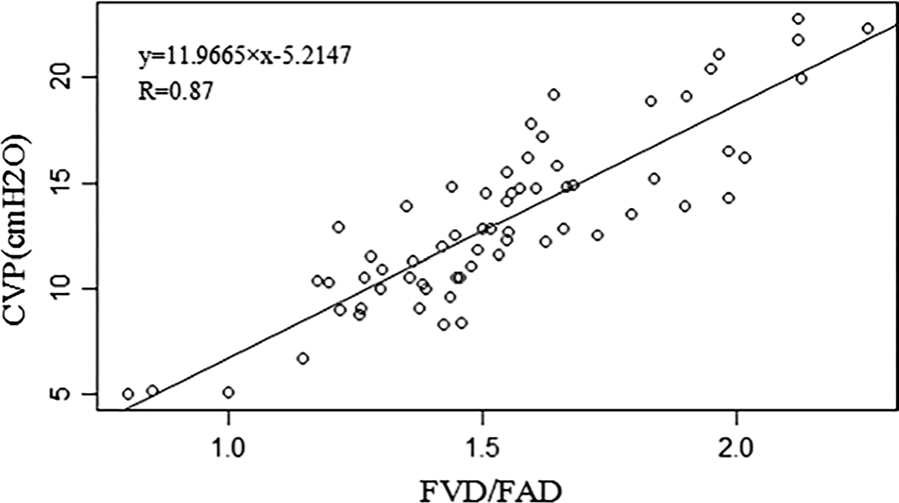
Correlation between the FVD/FAD ratio and CVP. *FVD* femoral vein diameter, *FAD* femoral artery diameter, *CVP* central venous pressure

Figure 2 shows the simple linear regression curve of the FVD/FAD ratio and CVP. Linear regression showed that the FVD/FAD ratio was correlated with CVP (R = 0.87, *P* < 0.0000). The following regression equation was obtained: CVP = 11.9665 × (FVD/FAD) − 5.2147 (F: 197.4, *P* < 0.0000). The adjusted R-square was 0.7542.

**Fig. 3:**
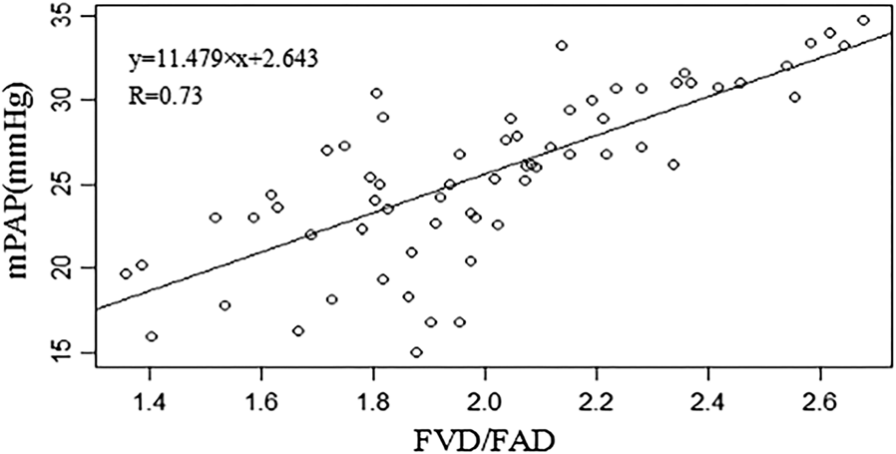
Correlation between the FVD/FAD ratio and mPAP. *FVD* femoral vein diameter, *FAD* femoral artery diameter, *mPAP* mean pulmonary artery pressure

Figure 3 shows the simple linear regression curve of the FVD/FAD ratio and mPAP. Linear regression showed that the FVD/FAD ratio was correlated with mPAP (R = 0.73, *P* < 0.0000). The following regression equation was obtained: mPAP = 11.479 × (FVD/FAD) + 2.643(F: 75.93, *P* < 0.0000). The adjusted R-square was 0.5393. However, when the FVD/FAD ratio was greater than 2.0, the adjusted R-square reached 0.6068.

**Fig. 4:**
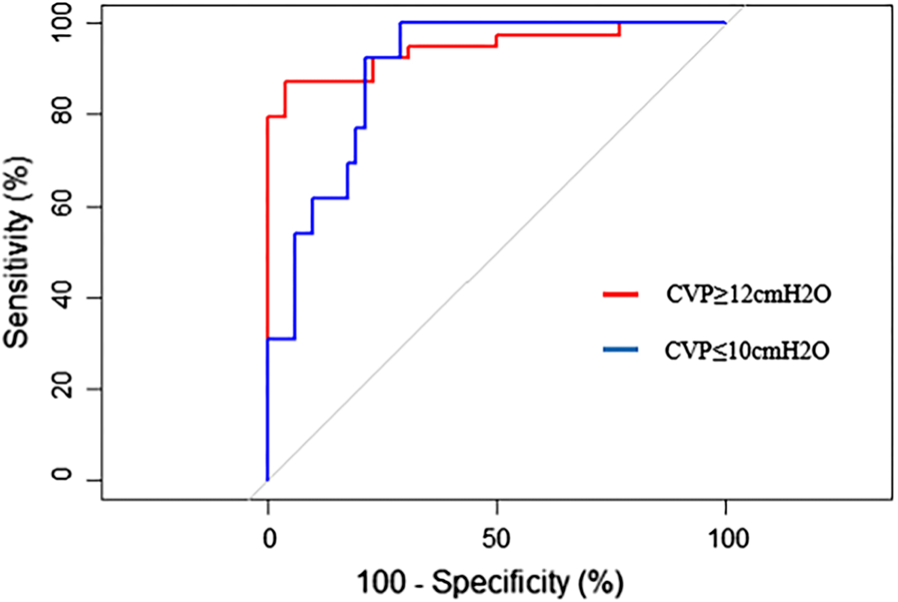
Receiver operating characteristic curve for prediction of various CVP values by the FVD/FAD ratio

Figure 4 shows the area under the curve (AUC) of various CVP values predicted by the FVD/FAD ratio. For prediction of CVP ≥ 12 cmH_2_O by the FVD/FAD ratio, the AUC was 0.945 (95% CI, 0.891–0.998). The best FVD/FAD ratio cut-off values were obtained according to the generated ROC curve. An FVD/FAD ratio ≥ 1.495 had the best test characteristics to predict a CVP ≥ 12 cmH_2_O (sensitivity 87%; specificity 96%; positive predictive value 97%; negative predictive value 83%). For prediction of CVP ≤ 10 cmH_2_O by the FVD/FAD ratio, the AUC was 0.896 (95% CI, 0.819–0.974). An FVD/FAD ratio ≤ 1.467 had the best test characteristics to predict a CVP ≤ 10 cmH_2_O (sensitivity 100%; specificity 71%; positive predictive value 46%; negative predictive value 100%). According to the simple linear regression curve of the FVD/FAD ratio and CVP, when the predicted CVP ≤ 5 cmH_2_O, the FVD/FAD ratio was ≤ 0.854.

**Fig. 5:**
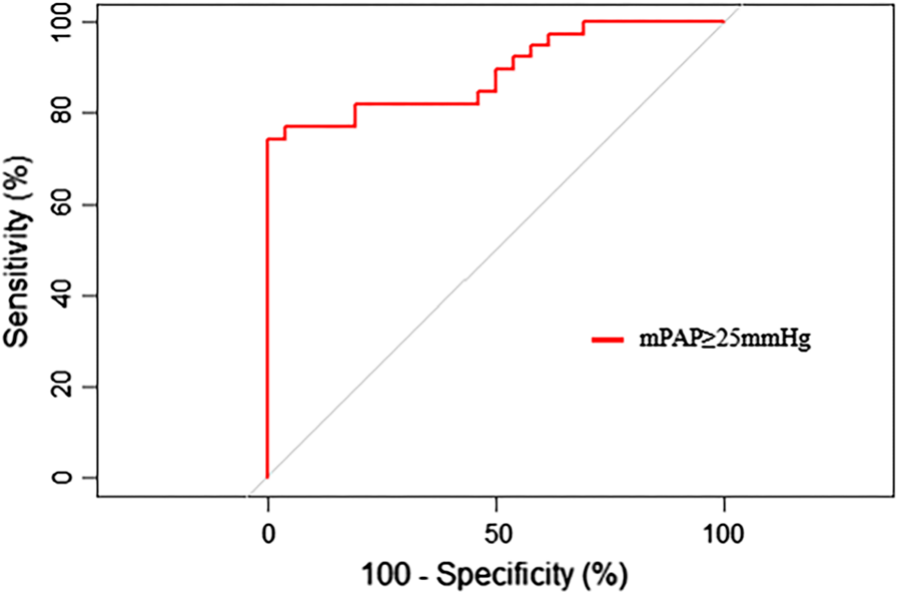
Receiver operating characteristic curve for prediction of mPAP value by the FVD/FAD ratio

Figure 5 shows the AUC of the mPAP value predicted by the FVD/FAD ratio. For prediction of mPAP ≥ 25 mmHg by the FVD/FAD ratio, the AUC was 0.889 (95% CI, 0.811–0.967). An FVD/FAD ratio ≥ 2.03 had the best test characteristics to predict an mPAP ≥ 25 mmHg (sensitivity 74%; specificity 100%; positive predictive value 100%; negative predictive value 87%).

## Discussion

Several studies have reported the evaluation of CVP by ultrasound measurement of the IVC or internal jugular vein (IJV) [17–20]. In a study by Nik Muhamad NA et al., the IVC diameter at end expiration was better at predicting CVP than the IJV height [21]. In a study by Prekker ME et al., the maximal IVC diameter was better at predicting CVP than the IJV aspect ratio [6]. However, it is a challenge to perform high-quality IVC measurements via ultrasound due to factors such as abdominal gas, abdominal dressings, or poor acoustic windows [22, 23]. Other studies have estimated mPAP by measuring the velocity of pulmonary regurgitation at the beginning of diastole, but this is difficult to measure, and changes in the structure of the right ventricle make it more difficult to measure [24, 25].

We performed baseline measurements in the normal population. Most of the blood in the normal human vascular system (approximately 65%) is in the veins, and most of the blood that flows to the lower extremities via the femoral artery returns almost equally to the femoral vein. This was also confirmed by the close equivalence of FVD and FAD under ultrasound observation in normal subjects. Therefore, it can be inferred that if the FVD is significantly greater than the FAD, it can prove venous system volume overload; if the baseline FVD is slightly smaller than the FAD and FV fails to expand effectively after coughing, it can prove that the patient's blood volume is low. Therefore, volume status can be easily, quickly and accurately judged by accurate measurement of the FVD and FAD. Femoral artery and vein are mayor vessels, running close to the skin even in overweight personssuperficial vessels, easy to measure under ultrasound. However, in patients with venous insufficiency, the volume of the femoral vein increased significantly [26], so we excluded the patients with venous insufficiency in this study.

In this study, we studied another non-invasive method for volume status assessment: the FVD/FAD ratio. CVP and mPAP were predicted by the FVD/FAD ratio. According to the R values obtained from this experiment, the FVD/FAD ratio was strongly correlated with CVP (R = 0.87) and mPAP (R = 0.73). In the study by Ciozda W et al., the authors summarized the correlation between the IVC diameter and the IVC-CI measured by ultrasound and CVP or right atrial pressure (RAP) that had been published in recent years [5]. The correlation coefficient R values between the ultrasound measurements of the maximum IVC diameter and CVP or RAP ranged from 0.35 to 0.86, with 57% having an R value between 0.5 and 0.78. The correlation coefficient R values between ultrasound measurements of IVC-CI and CVP or RAP ranged from − 0.27 to − 0.76, among which 62% were between − 0.5 and − 0.76. The correlation coefficient R between the FVD/FAD ratio measured by ultrasound and CVP in this experiment was 0.87. The FVD/FAD ratio was slightly better than the IVC in the correlation with CVP. The FVD/FAD ratio in this study was used to evaluate not only venous indicators but also arterial indicators.

In the simple linear equation established between the FVD/FAD ratio and CVP, the adjusted R-square was 0.7542, which means that 75.42% of the CVP variance was affected by the FVD/FAD ratio. In the simple linear equation of the FVD/FAD ratio and mPAP, the adjusted R-square was 0.5393, which means that 53.93% of the variance in mPAP was due to the FVD/FAD ratio. When the FVD/FAD ratio was greater than 2.0, the adjusted R-square was 0.6068, which means that 60.68% of the mPAP variance was due to the FVD/FAD ratio. In this study, we performed two sets of ROC analyses: (a) In this study, the best cut-off value of the FVD/FAD ratio for predicting a CVP ≥ 12 cmH_2_O was 1.495. The best cut-off value of the FVD/FAD ratio for predicting a CVP ≤ 10 cmH_2_O was 1.467. In previous studies, CVP ≤ 10 cmH_2_O was selected as the node for ROC curve analysis [6, 14]. Although there was a small number of CVP ≤ 5 cmH_2_O measurements, we determined that an FVD/FAD ratio ≤ 0.854 predicted a CVP ≤ 5 cmH_2_O through the simple linear regression curve of the FVD/FAD ratio and CVP. When FVD/FAD ratio ≤ 1, the mean exFVD/FAD ratio was 1.23 ± 0.10, which was far lower than the exFVD/FAD ratio of normal subjects. This suggests that the exFVD/FAD ratio is helpful for the diagnosis of hypovolemia. (b) In this study, the best cut-off value of the FVD/FAD ratio for predicting an mPAP ≥ 25 mmHg was 2.03. The specificity and positive predictive value reached 100%, indicating that when the FVD/FAD ratio was ≥ 2.03, all mPAP measurements according to the PAC were ≥ 25 mmHg. Although the FVD/FAD ratio in this study could not wholly and accurately predict mPAP, the FVD/FAD ratio could be a good screen for pulmonary hypertension.

There are some limitations in this study. First, we preferred high CVP groups in this study because some patients with hypovolemia were already receiving fluid therapy at the time of measurement. Therefore, there may be some deviations in the estimation and prediction of low CVP groups. Second, the sample size of our study was small, and the selected population was relatively homogeneous, so the results may not be representative of all populations. Therefore, we will increase the study of hypovolemic patients in the follow-up study to further verify the effectiveness of this method.

In conclusion, this study showed that the FVD/FAD ratio could accurately assess blood volume status and had certain clinical application value for diagnosing heart failure, assessing acute internal blood loss, diagnosing pulmonary hypertension, and so on.

## Conclusions

In this study, the measurement of the FVD/FAD ratio obtained via ultrasound was strongly correlated with CVP and mPAP. There was a linear relationship between the FVD/FAD ratio and CVP or mPAP. The exFVD/FAD ratio is helpful for the diagnosis of hypovolemia. Moreover, the FVD/FAD has a screening effect on patients with high mPAP.

## Data Availability

The datasets used and analysed during the current study are available from the corresponding author on reasonable request.

## Abbreviations

CVP: Central venous pressure
mPAP: Mean pulmonary artery pressure
FVD: Femoral vein diameter
FAD: Femoral artery diameter
MODS: Multiple organ dysfunction syndrome
PAC: Pulmonary artery catheter
ICU: Intensive care unit
IVC: Inferior vena cava
IVC-CI: Inferior vena cava collapsibility index
ROC: Receiver operating characteristic
AUC: Area under the curve
IJV: Internal jugular vein
RAP: Right atrial pressure
